# Supplementary material for: Interactive Structural Analysis of KH3-4 Didomains of IGF2BPs with Preferred RNA Motif Having m6A Through Dynamics Simulation Studies
Source: Int J Mol Sci. 2024 Oct 16;25(20):11118. doi: 10.3390/ijms252011118 (PMC11508745; doi:10.3390/ijms252011118)
Supplement: Supplementary file 1 [file ijms-25-11118-s001.zip › ijms-3167445-supplementary.pdf]

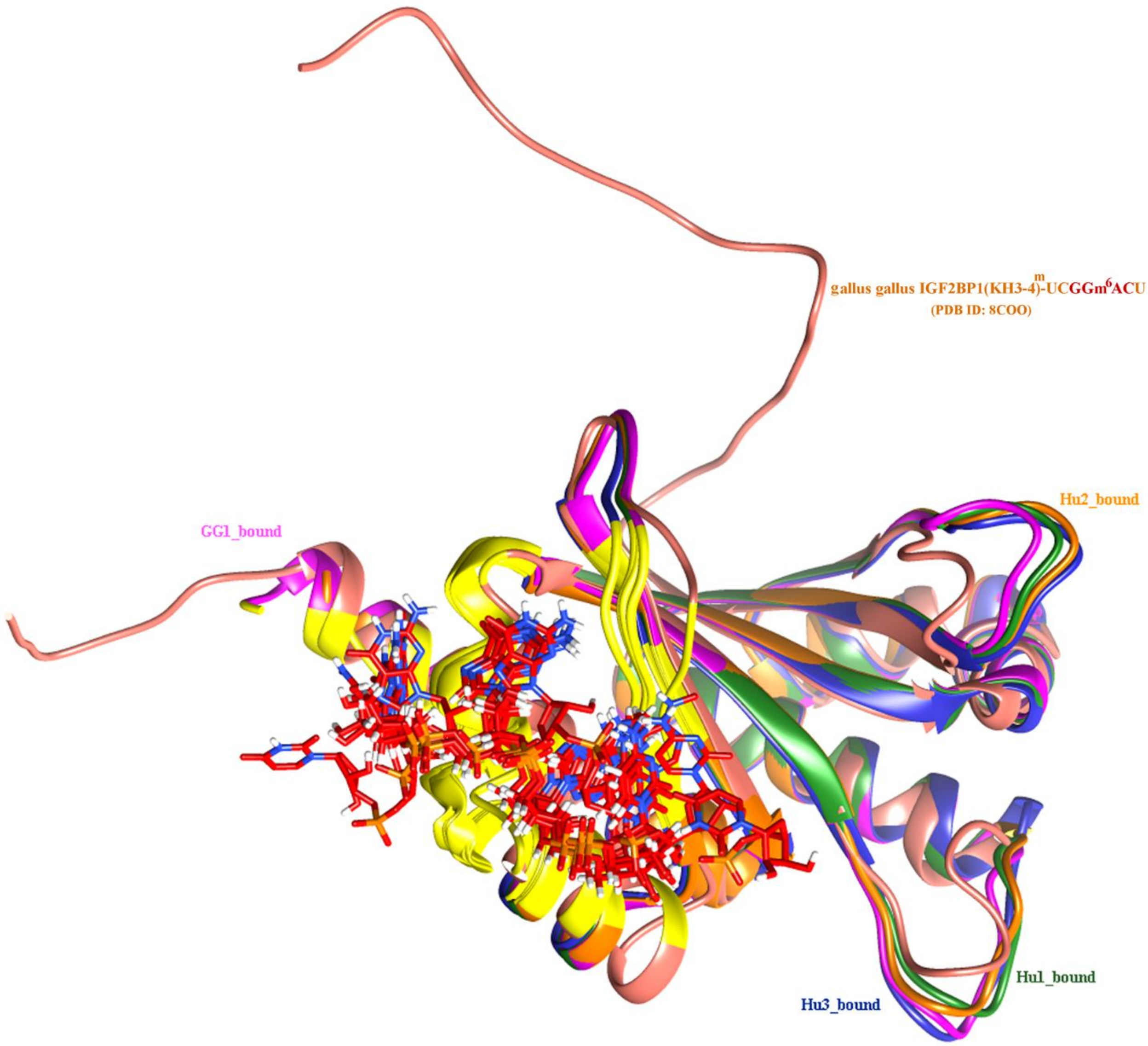

Figure S1. Pose orientation of superimposed experimental and docked complexes of IGF2BPs KH3-4 domains. Experimental gallus gallus IGF2BP1 (KH3-4)^{m}-UCGGm^{6}ACU complex (coral), gallus gallus GG1_bound (pink) and human Hu1-3_bound complexes are highlighted in green, orange and blue colors respectively. RNA motif in complexes is represented in stick with red color. The binding region of KH4 domain of all IGF2BPs are shown in yellow color.

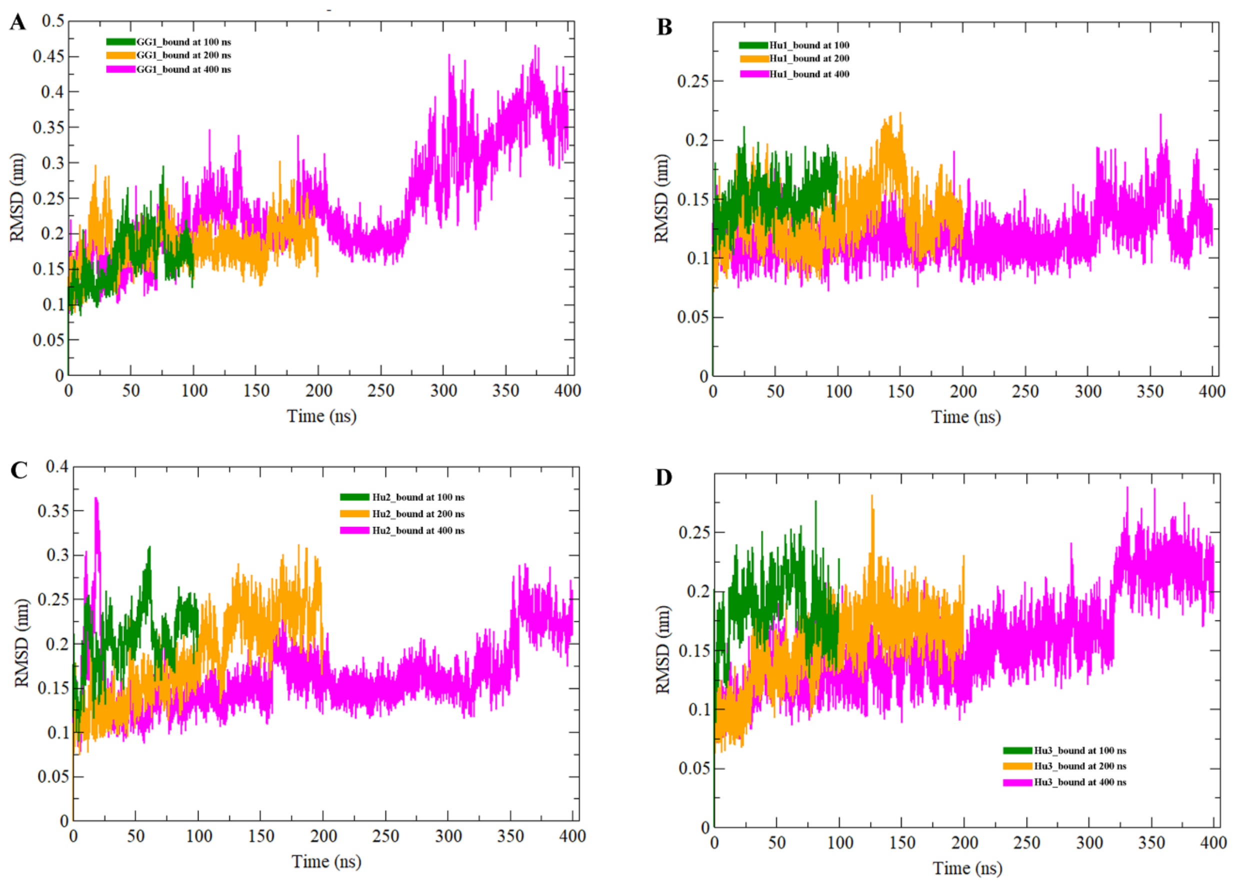

Figure S2. RMSD analysis of IGF2BPs (KH3-4) with GGm^{6}AC complexes stability across 100 ns, 200 ns, and 400 ns simulations. **(A)** gallus gallus GG1_bound stability over 100 ns, 200 ns, and 400 ns Simulations. **(B)**, **(C)** and **(D)** human Hu1-3_bound stability over 100 ns, 200 ns, and 400 ns Simulations.

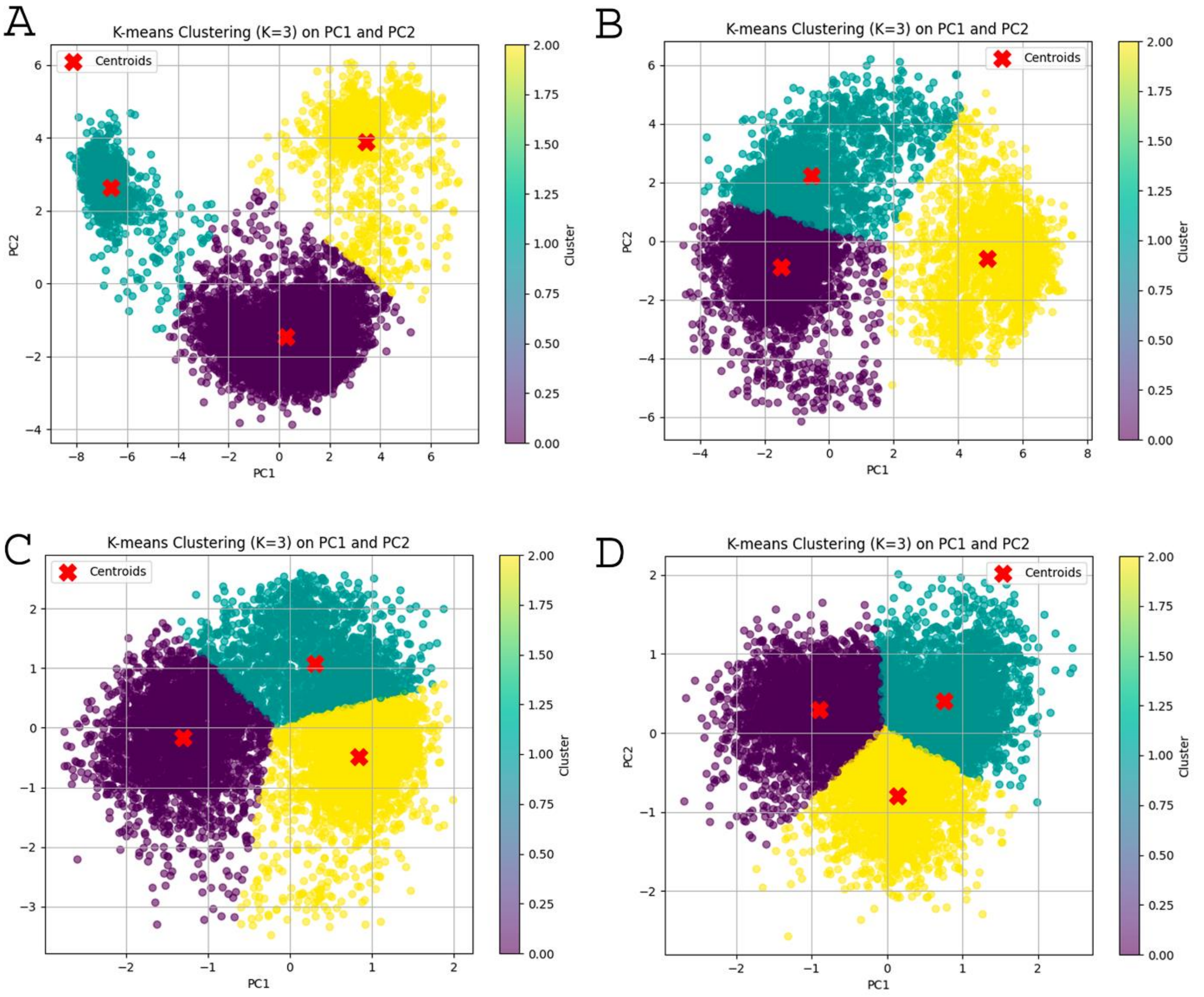

Figure S3. K-means Clustering (K=3) on PC1 and PC2 for Apo and Bound Complexes. Panel **(A)** apo_GG1, **(B)** GG1_bound, **(C)** apo_Hu1 and **(D)** Hu1_bound represent 2 D plots.

Table S1: Experimental 3D structures of KH3-4 domains of IGF2BPs and their complex with GGm^{6}AC available information.

**Type**	**Short code**	**Apo**	**Complex**
Gallus gallus IGF2BP1 (KH3-4)^m (PDB ID: 8COO)	GG1^m	✓	✓ UCGGm^{6}ACU with "GGm^{6}AC" RNA containing motif
Human_IGF2BP1(KH3-4) (PDB ID: 3KRM ; resolution: 2.75Å)	Hu1	✓	✗
Human_IGF2BP2 (KH3-4) (PDB ID: 6ROL; resolution: 2.10Å)	Hu2	✓	✗
Human_IGF2BP3 (KH3-4)	Hu3	✗	✗

Note: The crystal structure of gallus gallus IGF2BP1 (KH3-4) with mutations (KK422-423DD) bound to the RNA motif UCGGm^{6}ACU is available (PDB ID: 8COO), providing a well-characterized complex with the GGm^{6}AC RNA motif. The 3D experimental structures of KH3-4 domains of human IGF2BP1 (PDB ID: 3KRM ; resolution: 2.75Å) and IGF2BP2 (PDB ID: 6ROL; resolution: 2.10Å) were taken from www.rcsb.org/structure. Due to the absence of experimentally determined structure for the human KH3-4 domains of IGF2BP3, the full-length 3D structure of IGF2BP3 was retrieved using the AlphaFold database (AlphaFold ID: AF-O00425-F1) from the website (alphafold.ebi.ac.uk/entry/O00425). Finally cleaved the desired domains KH3-4 (405-553 AA) from full length Alphafold predicted 3D structure of IGF2BP3. Mutations (KK422-423DD) were introduced into the KH3 domain of all human IGF2BPs (KH3-4) and subsequently subjected to docking with the GGm^{6}AC RNA motif using the GRAMM and HDock web servers.

Table S2. Interaction of KH3-4 domains of human IGF2BP1-3 and gallus gallus IGF2BP1 with GGm^{6}AC RNA motif. (Hydrogen bonding residues are highlighted in bold).

**Organism**	**Complex**	**Short Code**	**Binding residues**	**HDock**	**GRAMM** (Dock Score)	**AlphaFold3** ipTM Score
Gallus gallus	IGF2BP1(KH3-4)-GGm^{6}AC	GG1_bound	Ala495,Ser496, Ala497, Ala498, Gly499, Arg500, Ile502, Gly503, Lys504, Gly505, Gly506, Val509, **Asn510,** Val520, Val521, **Val522**, Pro523, **Arg524**, Asp525, Gln526, Val534 and Val536	-144.6	190	0.93

Human	IGF2BP1(KH3-4)-GGm⁶AC	Hu1_bound	Ala495, Ser496, Ala497, Ala498, Gly499, Arg500, Ile502, Gly503, Lys504, **Gly505**, Gly506, Lys507, Thr508, Val509, Asn510, Gln513, Val520, Val521, Val522, Pro523, Arg524, Asp525, Gln526 and Val534	-138.4	187	0.92
Human	IGF2BP2(KH3-4)-GGm⁶AC	Hu2_bound	Ser495, Ser496, Thr497, Ala498, Gly499, Arg500, Ile502, Gly503, Lys504, **Gly505,** Gly506, Val509, Asn510, Gln513, Val520, Ile521, Val522, Pro523, Arg524, Asp525, Gln526, Val536 and Arg537	-152.3	220	0.90
Human	IGF2BP3(KH3-4)-GGm⁶AC	Hu3_bound	Ser495, Phe496, Ala497, Ala498, Gly499, Arg500, Ile502, Gly503, Lys504, **Gly505**, Gly506, Thr508, Val509, Asn510, Gln513, Val520, **Val521**, Val522, Pro523, **Arg524,** Asp525, Gln526, Val536 and Lys537	-140.6	180	0.93

Table S3. Interaction analysis of KH3-4 domains of human IGF2BP1-3 and gallus gallus IGF2BP1 with GGm⁶AC RNA motif at different MD simulation time scale. (Hydrogen bonding residues are highlighted in bold).

**Organism**	**Complex**	**Short Code**	**Binding residues**
Gallus gallus	IGF2BP1(KH3-4)-GGm⁶AC	GG1_bound	Ala498, Gly499, Ile502, Gly503, **Lys504**, **Gly505**, Gly506, Lys507, **Thr508**, Val509, **Asn510**, Val522, Pro523, Arg524, **Gln526** and Pro528
Human	IGF2BP1(KH3-4)-GGm⁶AC	Hu1_bound	Gly499, Arg500, Ile502, Gly503, Lys504, Gly505, Gly506, Val522, Pro523, **Arg524**, **Asp525, Gln526**, Val534

Human	IGF2BP2(KH3-4)-GGm⁶AC	Hu2_bound	Ser495 , Ala498, Gly499, , Ile502, Gly503, Lys504, Gly505, Gly506, , Val520, Ile521, Val522, Pro523, Arg524, **Asp525**, **Gln526**, Pro528 and Val534
Human	IGF2BP3(KH3-4)-GGm⁶AC	Hu3_bound	Ser495, Phe496, Ala498, Gly499, Ile502, Gly503, Lys504, **Gly505**, Gly506, Val521, Val522, Pro523, Arg524, Gln526, Val534

Note: These are the interaction analysis of all complexes after MD Simulation. We observed that gallus gallus IGF2BP1(KH3-4)-GGm^{6}AC (GG1_bound) and human IGF2BP1(KH3-4)-GGm^{6}AC (Hu1_bound) showed the stability throughout 400 ns MD simulation time scale, whereas IGF2BP2(KH3-4)-GGm^{6}AC (Hu2_bound) and IGF2BP3(KH3-4)-GGm^{6}AC (Hu3_bound) remained stable at KH4 domain till 150 ns and 120 ns time scale respectively and after moving toward the KH3 domain. We have mentioned the residues of interaction in this Table S3.

Table S4. MD simulation with stability results of RNA motif GGm^{6}AC with KH4 domain

**Organism**	**Complex**		**100 ns**	**200 ns**	**400 ns**
Gallus gallus	IGF2BP1(KH3-4)-GGm^{6}AC	GG1_bound	Stable	Stable	stable
Human	IGF2BP1(KH3-4)-GGm^{6}AC	Hu1_bound	Stable	Stable	stable
Human	IGF2BP2(KH3-4)-GGm^{6}AC	Hu2_bound	Stable	Unstable	Unstable
Human	IGF2BP3(KH3-4)-GGm^{6}AC	Hu3_bound	Stable	Unstable	Unstable
